# Exploring theory of mind abilities in Lebanese chronic patients with schizophrenia: A cross-sectional study^☆^

**DOI:** 10.1016/j.scog.2025.100385

**Published:** 2025-08-06

**Authors:** Marie Ghosn, Chadia Haddad, Jean-Marc Rabil, Georges Haddad

**Affiliations:** a Department of Psychology, University of Balamand Kalhat, Al Koura, P.O box 100, Tripoli, Lebanon; b Research Department, Psychiatric Hospital of the Cross, Jal Eddib, Lebanon; c INSPECT-LB (Institut National de Santé Publique, d'Épidémiologie Clinique et de Toxicologie-Liban), Beirut, Lebanon; d Inserm U1094, IRD UMR270, Univ. Limoges, CHU Limoges, EpiMaCT - Epidémiologie des maladies chroniques en zone tropicale, Institut d'Epidémiologie et de Neurologie Tropicale, OmegaHealth, Limoges, France; e Faculty of Public Health, Lebanese University, Fanar, Lebanon; f School of Medicine and Medical Sciences, Holy Spirit University of Kaslik, P.O box 446, Jounieh, Lebanon

**Keywords:** Social cognition, Theory of mind, Mentalizing, Neuro-cognition, Schizophrenia, Depression, Insight, False belief task

## Abstract

**Introduction:**

In recent decades, social cognition has become a central focus in schizophrenia research. Multiple previous studies reported impairments in multiple social cognitive domains. One particular domain of social cognition affected in schizophrenia is “Theory of Mind (TOM). The objective of this study was to examine the heterogeneity of ToM impairments within the schizophrenia population and compare TOM performance between individuals diagnosed with schizophrenia and a matched healthy control group.

**Methods:**

a cross-sectional study was conducted between June 2024 and September 2024 at the Psychiatric Hospital of the Cross (HPC) in Lebanon that explored TOM abilities in 146, chronic Lebanese schizophrenia inpatients and 50 healthy controls, using the Arabic translation of the “TOM-15,” a False Belief Task.

**Results:**

A significant difference between schizophrenia patients and healthy controls was found, with the patient group scoring poorer on both first-order, and second-order false belief and in the comprehension control task of the TOM-15. Furthermore, among the patient population, impairments in second-order false belief were more pronounced than first-order. Performance on the BACS scale for neuro-cognition showed moderate associations with performance on the TOM-15. Multivariable analysis revealed a negative association between depression and second-order tasks as well as females outperforming males in TOM-15, especially in the second-order task.

**Conclusion:**

The results revealed significant TOM impairments in patients with schizophrenia as compared to healthy controls, with greater difficulties observed in second-order false belief tasks.

## Highlights

• Lebanese chronic inpatients exhibit moderate impairments in Theory of Mind
• Higher depressive symptoms were associated with poorer Theory of Mind
• Theory of Mind was found to be weakly correlated with neurocognitive functioning.
• The clinical symptoms and theory of mind did not significantly correlate.

## 1 Background

Schizophrenia is a chronic psychiatric disorder characterized by a spectrum of behavioral, emotional and cognitive dysfunctions that manifest during late adolescence and early adulthood. It affects approximately 24 million individuals globally, accounting for 0.32 % of the population, or 1 in 300 people (Charlson et al., 2018). In the context of Lebanon, where the population exceeds five million, including both native and displaced individuals, the Global Health Observatory (GHO) estimates that around 50,000 individuals live with a psychotic illness (El-Khoury et al., 2018). However, despite its relatively low prevalence rate, schizophrenia presents as a multifaceted clinical challenge associated with a significant disability burden on an individual, interpersonal, and community level.

Extensive research over the past decades has significantly advanced our understanding of the disorder's pathophysiology, alongside the management of its positive symptoms (Harvey, 2013). However, in recent years, there has been a notable shift in schizophrenia research towards investigating other central domains of the disorder, with a specific emphasis on cognition, particularly, social cognition. Social Cognition refers to the mental processes involved in perceiving, interpreting, and responding to the intentions, dispositions, and behaviors of others (Green et al., 2015). It supports essential interpersonal functions, such as recognizing emotions, understanding nonverbal cues, interpreting spoken language, and inferring others' mental states (Harvey, 2013). Impairments in social cognition have been long recognized as a fundamental feature of schizophrenia. Studies have reported impairments across multiple social cognitive domains, including e*motion processing*, which involves recognizing and interpreting emotional states in oneself and others, *attributional style or bias*, refers to how individuals explain the causes of social events or behaviors, *social perception*, or the ability to interpret social cues and contexts and lastly, *Theory of Mind* (TOM) *“Mentalizing” “Meta representation” “Metacognition”*, the ability to attribute mental states, such as beliefs, desires, and intentions, to oneself and others (Premack and Woodruff, 1978).

Among the domains of social cognition, TOM has received particular attention in clinical research due to its contribution to the social difficulties observed across various mental disorders. The investigation of TOM deficits in schizophrenia was first initiated by Frith (1992) (Frith, 1992; Frith, 2000). The motivation behind Frith's investigation stemmed from the growing recognition that schizophrenia, a disorder traditionally viewed in terms of its positive and negative symptoms, also involved disruptions in how individuals perceive and interact with others. Frith's hypothesis posited that many of the central symptoms of the disorder, including delusions, hallucinations, and social dysfunction, could be attributed to disturbances in cognitive functions related to the understanding of one's own mental state and the mental states of others. In fact, he conceptualized schizophrenia as “a disorder of representation of mental states” (Frith, 2015). Since Frith's proposal, the relationship between TOM in schizophrenia has become a significant area of research. This has resulted in a substantial body of literature reporting deficits in the overall TOM ability in individuals with schizophrenia, demonstrating that on average, their TOM performance was more than one standard deviation lower than that of healthy controls (Harvey, 2013; Pinkham, 2014; Harrington et al., 2005; Brüne, 2005; Sprong et al., 2007).

Various cognitive measures have been developed over the years to assess TOM abilities, with the “False Belief Task” being the most frequently used (Baron-Cohen, 1999). This measure incorporates tasks that assess first-order and second-order false belief/deception abilities. First-order tasks involve understanding that individuals can act based on beliefs or deceptions that do not align with reality. Second-order tasks require participants to recognize that one person can have a belief about another person's belief, even if that belief may not align with reality (Harrington et al., 2005; Bora et al., 2008). Impairments have been documented across both first- and second-order levels in individuals with schizophrenia, though the severity and pattern of these deficits vary across studies (Harrington et al., 2005; Sprong et al., 2007). Several studies have reported more pronounced impairments in second-order TOM, which is often attributed to its reliance on higher-order neurocognitive processes (Corcoran et al., 1995; Pickup and Frith, 2001; Wu et al., 2023). Interestingly, other studies have documented adequate performance in second-order tasks despite marked deficits in first-order, which challenges the assumption of a hierarchical structure in TOM processing (Stratta et al., 2011; Ho et al., 2015).

Moreover, TOM has long been recognized as a multidimensional construct. Over time, research has shown that TOM is not a unitary function, but rather involves several components that work together to enhance the understanding of others mental states (Pinkham, 2014; Green et al., 2008). These components reflect both the cognitive and emotional significance of the intentions of others. *Cognitive Theory of Mind* involves understanding others' beliefs, intentions, knowledge, and perspectives, enabling individuals to make inferences about what others think or know in specific situations. In contrast, *affective Theory of Mind* involves recognizing and responding to the emotions of others (Brothers and Ring, 1992). This distinction has clinical relevance, as different TOM components may be differentially affected in schizophrenia. For instance, deficits in cognitive TOM have been associated with positive symptoms, such as delusions and hallucinations (Montag et al., 2011). In contrast, impairments in affective TOM appear to be associated to negative symptoms, including social withdrawal and blunted affect (Wu et al., 2023; Shamay-Tsoory et al., 2007). However, only a limited number of studies have examined both components within the same experimental paradigm (Wu et al., 2023; Okruszek et al., 2018). Among the limited studies that have done so, Okruszek et al. (2018) reported more pronounced impairments in cognitive TOM. These reported impairments may be partially attributed to underlying neurocognitive impairments such as, working memory and executive dysfunction (Okruszek et al., 2018).

Research examining the relationship between clinical symptomatology and TOM in individuals with schizophrenia have reported considerable heterogeneity. While several studies have reported significant associations between specific symptom clusters and TOM performance, these relationships have lacked consistency across the literature. For example, some studies have found that negative symptoms, are strongly correlated with deficits in second-order TOM tasks (Corcoran et al., 1995; Pickup and Frith, 2001). Others have linked disorganized symptoms, to difficulties in integrating contextual cues necessary for interpreting others' mental states (Corcoran et al., 1995; Sarfati and Hardy-Baylé, 1999). Studies exploring the relationship between positive symptoms, and TOM performance have yielded mixed results, with some identifying correlations and others reporting no significant associations (Harrington et al., 2005; Abdel-Hamid et al., 2009). In contrast, a number of studies have not identified any significant associations between specific symptom dimensions and TOM performance (Ho et al., 2015; Okruszek et al., 2018; Fernandez-Gonzalo et al., 2013; Wang et al., 2013; Li et al., 2017).

The relationship between TOM impairments and neurocognitive functioning in schizophrenia has been the focus of extensive research, yielding mixed findings and leading to two main hypotheses. Frith and Frith (2003) proposed that TOM deficits in schizophrenia are independent of general cognitive functioning, suggesting that difficulties in understanding others' mental states arise from mechanisms distinct from broader cognitive processes (Frith and Frith, 2003). Studies supporting this hypothesis have reported persistent TOM impairments even after statistically controlling for general cognitive deficits (Corcoran et al., 1995; Pickup and Frith, 2001; Frith and Frith, 2003). Conversely, other studies have argued that, TOM impairments are partially dependent on executive functioning, indicating that these deficits may be linked to challenges in processing contextual information (Fernandez-Gonzalo et al., 2013; Greig et al., 2004; Ventura et al., 2013; Mazza et al., 2012; Thibaudeau et al., 2020). Empirical support for this view includes several studies demonstrating significant associations between neurocognitive and TOM performance. For instance, Fanning et al. (2012) demonstrated that neurocognitive abilities account for approximately 19 % of the variance in TOM performance among individuals with schizophrenia (Fanning et al., 2012). This finding shows that individuals with neurocognitive impairments are more likely to also experience difficulties with TOM (Thibaudeau et al., 2020; Fanning et al., 2012).

The potential effects of antipsychotic medication on social cognition, particularly Theory of Mind (ToM), were examined. Emerging evidence suggests that second-generation antipsychotics (SGAs) may be more effective than first-generation antipsychotics (FGAs) in enhancing ToM performance in individuals with schizophrenia (Kucharska-Pietura and Mortimer, 2013; Giralt-López et al., 2024). For instance, Zhong et al. (2021) found that paliperidone significantly improved performance across multiple ToM tasks, whereas haloperidol showed benefits only on the simplest task (Zhong et al., 2021). Similarly, Mizrahi et al. (2007) reported ToM improvement over six weeks, primarily among patients treated with SGAs (Mizrahi et al., 2007). Additionally, in recent years, there has been a lot of interest in studying the relationship between sociodemographic factors and TOM (Clutterbuck et al., 2023). Age, marital status, and educational level have all been found to be possible determinants of TOM performance (Clutterbuck et al., 2023). According to some research, older individuals' capacity to infer the mental states of others may deteriorate as a result of age-related changes in cognitive functioning (Gigi and Papirovitz, 2025; Cho and Cohen, 2019). Improved cognitive and metacognitive abilities, which are essential for comprehending and predicting the intents and feelings of others, are frequently linked to higher education levels (Li et al., 2013). Additionally, via greater social interaction and chances for perspective-taking, married status, especially being in a stable relationship, may improve TOM (Haghighi et al., 2024). Collectively, these results highlight the complex interplay between social-cognitive performance and personal life conditions.

From a cultural perspective, it has been proposed that TOM can be an acquired skill that develops through social learning and environmental exposure (Lillard, 1998). According to this view, individuals gradually learn to interpret others' mental states such as, beliefs, intentions, and emotions, through culturally specific norms, social practices, and communication styles (Heyes and Frith, 2014). However, it remains unclear whether TOM is a cognitive capacity expressed similarly across cultures, or whether its development and expression are shaped by specific cultural contexts. In schizophrenia, cross-cultural research on TOM impairments is notably scarce, with only a small number of studies conducted to date and limited theoretical development in the field. Although some findings suggest comparable deficits across different cultural groups (Lee et al., 2018), other studies have observed variation in performance on more complex TOM tasks, such as second-order false belief reasoning, indicating that cultural factors may influence how these impairments manifest (Beck et al., 2020).

However, despite the increasing body of literature exploring social cognition in schizophrenia across various global contexts, there remains a significant gap in research on specific domains of social cognition, particularly TOM abilities, within the Arab world. More specifically, no studies have yet comprehensively explored TOM impairments in schizophrenia patients in Lebanon, leaving a critical gap in understanding how these cognitive deficits manifest in this population.

Thus, addressing this gap is crucial not only for advancing our knowledge of the characteristics and extent of TOM deficits in Lebanese patients with schizophrenia, but also to establish normative data for assessing social cognitive functioning within this cultural context. Additionally, such efforts can help facilitate the development of contextually adapted interventions and support the inclusion of Lebanese populations in prospective international studies.

Given this crucial gap, the primary objective of this study was to investigate TOM impairments in individuals with schizophrenia, both in terms of the heterogeneity within the patient population and in comparison, to a matched healthy control group. To achieve this, we utilized the Arabic translation of the TOM-15, a cognitive False Belief Task, to assess TOM abilities. Furthermore, the study examined the potential influence of clinical symptomatology and neurocognitive functioning, alongside other variables that prior research has identified to be associated with TOM performance. These variables included depressive symptoms (Nestor et al., 2022), insight into illness (Ng et al., 2015), type and dosage of antipsychotic medication (Desgranges et al., 2012), as well as demographic factors such as age, gender, education level, and marital status (Abu-Akel and Bo, 2013; Canuso and Pandina, 2007).

## 2 Methods

### 2.1 Study design and participants

This cross-sectional study was conducted at the Psychiatric Hospital of the Cross (HPC) in Lebanon between June and September 2024. The sample consisted of 146 chronic inpatients diagnosed with schizophrenia or schizoaffective disorder and 50 demographically matched healthy controls. Inclusion criteria for the patient group required a confirmed DSM-5 diagnosis of schizophrenia or schizoaffective disorder, established through consensus by a multidisciplinary panel of psychiatrists and licensed clinical psychologists. Patients were also required to demonstrate clinical stability, as having received consistent antipsychotic treatment for a minimum of six months, with no dosage adjustments in the three months preceding enrollment (Leucht et al., 2012). Additional eligibility criteria included fluency in Arabic and an age range of 18 to 65 years.

Exclusion criteria for the patient group included a history of traumatic brain injury, stroke, or other neurological conditions such as epilepsy, as well as substance use disorder or developmental disabilities, as these factors could confound the assessment of cognitive impairments associated with schizophrenia. For the control group, exclusion criteria included a history of brain injury, neurological disorders, personal or family history of psychiatric illness, or substance use or dependence, with the exception of nicotine dependence.

**Fig. 1:**
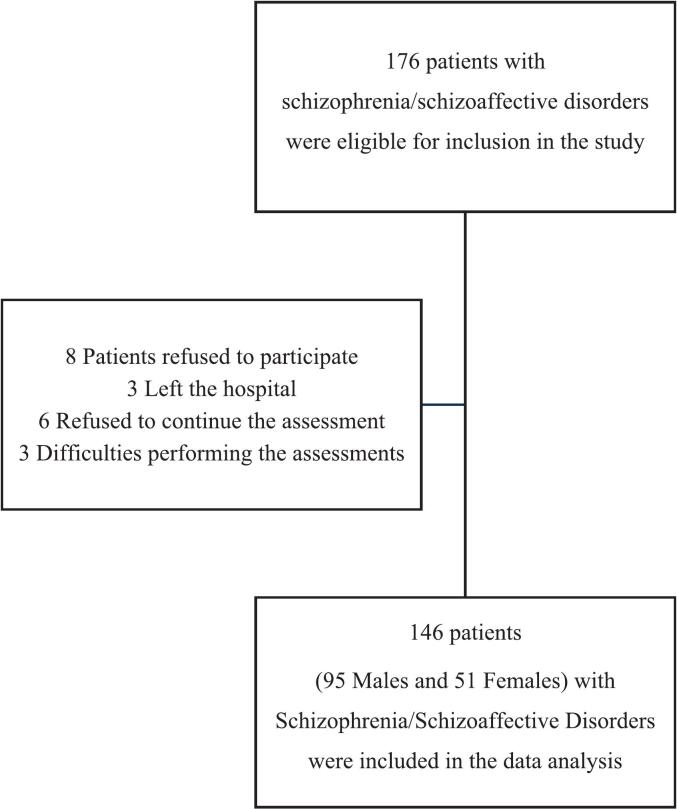
Enrollment of patients.

A total of 176 patients were initially selected based on the inclusion criteria. Of these, 146 patients (95 males and 51 females) were included in the study. 20 patients (11 males and 9 females) were excluded for the following reasons: 8 declined to participate, 3 left the hospital, 6 discontinued the assessment, and 3 experienced difficulties with the assessments (Fig. 1).

### 2.2 Ethical approval and procedure

Ethical approval for this study was obtained from both the Ethics and Research Committee of the Psychiatric Hospital of the Cross (reference number: HPC-001-04-24), in accordance with the hospital's Regulatory Research Protocol, and the Institutional Review Board of the Faculty of Arts and Sciences at the University of Balamand (reference number: IRB-REC/o/024-25/0624), ensuring compliance with established ethical research standards.

Participants were provided with a detailed explanation of the study's objectives and procedures, and written informed consent was secured prior to their participation. They were made aware of their right to discontinue their involvement at any time without consequences. Additionally, data collection was conducted in a private and secure environment within the psychiatric hospital, ensuring confidentiality and creating a comfortable setting for participants.

Three academic and clinical backgrounds in psychology or psychiatry conducted the assessments. To promote consistency and reliability in the administration of assessment tools, each examiner underwent both individual and group training sessions covering the full battery of measures. In addition, data collection was conducted under the supervision of the lead researcher, who provided structured feedback to ensure uniformity in administration. The entire evaluation process lasted approximately 60 to 90 min per participant, depending on individual pace and clinical factors. To minimize fatigue and optimize participant engagement, scheduled breaks of approximately five minutes were provided between assessment tasks.

### 2.3 Materials

The assessments were conducted in Arabic, the native language of Lebanon. The first part of the evaluation involved a sociodemographic questionnaire, which collected information on participants' demographic and clinical characteristics. This included age, gender, education level, marital status, family history of mental disorders, schizophrenia subtype, duration of hospitalization, length of illness, and number of hospital admissions.

The second part of the evaluation included the following measures:

Theory of Mind-15 (TOM-15)

The TOM-15 is a cognitive false belief assessment consisting of 15 narratives, including eight first-order and seven second-order false belief stories. It is divided into two components: a false belief task and a comprehension task, both utilizing the same narratives but with different questions. The stories depict everyday situations in which a character forms an incorrect belief about reality. Some stories are original, while others are adapted from existing literature in developmental psychology (Desgranges et al., 2012).

Each story is presented in three sequential parts on a single board, accompanied by color illustrations, and a verbal caption to minimize the influence of potential confounding cognitive processes that may interfere with ToM performance. The TOM-15 was translated into Modern Standard Arabic, and each story was printed separately and administered according to standardized instructions. Responses were recorded on a scoring sheet (Desgranges et al., 2012).

Scoring involves determining the number of correct responses for first-order (maximum score = 8) and second-order (maximum score = 7) questions. After completing the false belief task, participants revisit all 15 narratives and answer a comprehension question for each story to assess general understanding. The total scores for the false belief and comprehension tasks are obtained by summing the number of correct responses, each with a maximum score of 15 (Desgranges et al., 2012). The Cronbach's alpha value for the false belief task was 0.777, and for the comprehension task was 0.794.

The Brief Assessment of Cognition in Schizophrenia (BACS)

The BACS is a neuropsychological battery, recently validated in Lebanon (Haddad et al., 2021), used to evaluate cognitive functioning in patients with schizophrenia (Keefe et al., 2004). It consists of six sub-scales, including list learning (verbal memory), digit sequencing (working memory), token motor task (psychomotor function), semantic fluency (verbal fluency), symbol coding (attention and speed of information processing), and Tower of London (executive function) (Keefe et al., 2004). The Cronbach's alpha value for the total BACS scale was 0.880.

### 2.4 Clinical measures

#### 2.4.1 Positive and Negative Syndrome Scale (PANSS)

The PANSS is a scale validated in Lebanon among patients with schizophrenia (Hallit et al., 2017). It consists of 30 items, divided into 3 subcomponents: positive symptoms (seven items: P1-P7), negative symptoms (seven items: N1- N7) and symptoms of general psychopathology (16 items: G1-G16). All items are scored with values from 1 to 7, with 1 reflecting no symptoms and 7 reflecting extremely severe symptoms. The scores of these scales are calculated by the sum of the elements of each component. The score for the positive and negative scales varies between 7 and 49 while the score varies from 16 to 112 for the general psychopathology scale. The total score is obtained by the sum of all the items (Kay et al., 1987). The Cronbach's alpha value was 0.888 for the total PANSS scale, 0.798 for the positive subscale, 0.775 for the negative subscale, and 0.838 for the general psychopathology subscale.

#### 2.4.2 Calgary Depression Scale for Schizophrenia (CDSS)

The CDSS is designed for the assessment of depression in schizophrenia. It includes nine questions regarding depression, self-deprecation, hopelessness, thoughts of guilt, morning sadness, early waking, suicide and observed depression. The response score ranges from 0 to 3 with 0 meaning no symptoms and 3 meaning the most severe symptoms. The total score of these nine questions would indicate the presence or absence of a depressive episode (Addington et al., 1993). The Cronbach's alpha value in the current study was 0.805.

#### 2.4.3 Insight Scale for Psychosis (IS)

It is a self-assessment scale for insight, made up of eight items divided into three subscales. The insight scale allows us to observe the patient's level of general awareness of their mental health. This scale makes it possible to measure the score of three subscales: awareness of illness (total score of items 1 and 8); awareness of symptoms (total score of items 2 and 7); awareness of the need for treatment (total score of items 3, 4, 5 and 6 (the items must be added and divided by 2). Each subscale has a total score that ranges from 1 to 4 (3 or 4 = good insight, 1 or 2 = poor insight) (Birchwood et al., 1994). The Cronbach's alpha value in the current study was 0.767.

### 2.5 Data analysis

The statistical data in this study was analyzed using the Statistical Package for the Social Sciences (SPSS) software version 25. Descriptive statistics were first calculated, with categorical variables expressed as counts and percentages, and continuous variables reported as means and standard deviations. To assess the distribution of the sample, normality was checked through visual inspection of histograms, and skewness and kurtosis values were found to be within the acceptable range (below |1.96|), confirming a normal distribution. Further verification of normality was conducted using the normality line on the regression plot and scatter plots of the residuals.

For group comparisons, an independent-sample *t*-test was applied to examine differences in TOM performance between two independent groups (healthy control group and the patient group). Analysis of variance (ANOVA) test was used to compare performance on ToM tasks, specifically first-order false belief, second-order false belief, and comprehension across three education level groups. Pearson correlation was used to assess the linear relationships between continuous variables, and ToM scores.

A multivariable linear regression analysis was performed among patients with schizophrenia to examine the associations between demographic, clinical, and cognitive variables and TOM subtest scores. Three separate models were conducted, with first-order false belief, second-order false belief, and comprehension scores as the dependent variables. Independent variables included age, sex, marital status, education level, family history of psychiatric illness, depression scale, insight scale, PANSS subscales, and BACS total score.

A Bonferroni correction was performed to correct the *p*-value over the multiple comparisons between the predictor variables and the TOM tets. Since 21 comparisons were made, the formula for the Bonferroni corrected/adjusted p-value was α/21 = 0.05/21 = 0.002. All statistical tests were considered significant at a p-value of less than 0.002.

## 3 Results

### 3.1 Sample description

Sociodemographic characteristics of the participants are described in Table 1. Mean age of patients with schizophrenia was 52.40 ± 7.35 years, with 65.1 % males. The majority (93.8 %) were single, 42.5 % had a complementary level of education and 36.8 % had a family history of psychiatric illness. Also, 51.5 % have a paranoid disorder and the mean duration of illness and hospitalization were 25.89 ± 9.68 and 14.92 ± 9.09 years respectively. The mean chlorpromazine equivalent dose was 1066.36 ± 921.73.

**Table 1:** Sociodemographic and clinical characteristics of the total sample (*N* = 196).

	Schizophrenia patients (*N* = 146)	Healthy control (*N* = 50)	p-value
	Frequency (%)	Frequency (%)	
Sex			
Male	95 (65.1 %)	26 (52.0 %)	0.101
Female	51 (34.9 %)	24 (48.0 %)	

Education level			
Complementary	62 (42.5 %)	11 (32.4 %)	0.355
Secondary	66 (45.2 %)	16 (47.1 %)	
University	18 (12.3 %)	7 (20.6 %)	

Marital status			
Single/widowed/divorced	137 (93.8 %)	23 (46.0 %)	**<0.001**
Married	9 (6.2 %)	27 (54.0 %)	

Family history of psychiatric illness			
Yes	53 (36.8 %)	4 (11.8 %)	0.005
No	91 (63.2 %)	30 (88.2 %)	

Diagnostic (DSM- V)			
Paranoid	70 (51.5 %)		
Disorganized	9 (6.6 %)		
Undifferentiated.	22 (16.2 %)		
Residual	4 (2.9 %)		
Schizoaffective	31 (22.8 %)		


	Schizophrenia patients (*N* = 146)	Healthy control (*N* = 50)	p-value
	Mean ± SD	Mean ± SD	
Age	52.40 ± 7.35	51.74 ± 7.58	0.636
Duration of illness in years	25.89 ± 9.68		
Duration of hospitalization in years	14.92 ± 9.09		
Chlorpromazine equivalent dose	1066.36 ± 921.73		

Values marked in bold indicate significant p-values after Bonferroni correction (p<0.002).

The healthy control group was matched with the schizophrenia group according to sex, education level, and age. The two groups differ in marital status and family history of psychiatric illness. Married participants without any psychiatric illness were found in the control group as compared to the patient group.

### 3.2 Description of the scales used

Table 2 describes the medians, means, SD, and ranges of the scales used in this study among patients with schizophrenia. All means and medians were moderate to low. The lowest mean according to the maximum level was found in the depression and insight scale.

**Table 2:** Description of the quantitative scales among patients with schizophrenia.

	Mean ± SD	Median	Minimum	Maximum
Theory of Mind (TOM-15)				
First order false belief	4.43 ± 2.11	5.00	0	8.00
Second order false belief	3.90 ± 1.87	4.00	0	7.00
Total false belief	7.94 ± 3.62	8.00	0	15.00
Comprehension	10.63 ± 3.52	11.00	0	15.00
PANSS total score	75.86 ± 25.99	71.00	30.00	147.00
Positive	17.70 ± 8.84	16.00	7.00	49.00
Negative	17.30 ± 7.81	16.00	7.00	44.00
General psychopathology	40.86 ± 15.37	39.00	16.00	78.00
Insight scale	5.28 ± 2.14	5.50	0	11.00
Calgary scale	4.85 ± 4.51	4.00	0.00	22.00

### 3.3 Bivariate analysis

**Fig. 2:**
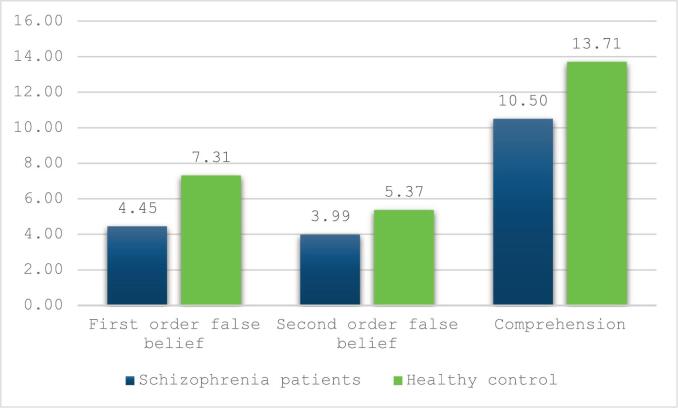
Adjusted mean of the TOM-15 scale between patients with schizophrenia and health control, after adjusting for potential confounders (age, gender, marital status, education level and family history of psychiatric illness) (*p* < 0.05 for all).

Table 3 presents the bivariate analysis of Theory of Mind (TOM-15) scores. Healthy controls demonstrated significantly higher first-order (*p* < 0.001), second-order (p < 0.001), and comprehension scores (p < 0.001) compared to individuals with schizophrenia (Fig. 2). schizophrenia patients scored significantly lower than healthy controls on the total BACS score as well as on all individual cognitive domains, including verbal memory, working memory, motor speed, verbal fluency, attention and speed of information processing, and executive function (all *p*-values <0.001) (Supplementary Table S1). After bonferroni correction no significant associations were observed for sociodemographic characteristics and TOM tasks (*p* > 0.002 for all). Age showed a weak, non-significant negative correlation with comprehension (*r* = −0.164, *p* = 0.053).

### 3.4 Correlational analyses

Table 4 presents the correlation analysis between TOM and quantitative scales among patients with schizophrenia. Significant positive correlations were found between ToM subtests and the BACS total score (first-order: *r* = 0.318, *p* = 0.001; comprehension: *r* = 0.331, *p* = 0.002). Among BACS subtests, attention/speed of information processing were significantly associated with comprehension TOM15 test (*r* = 0.362, *p* = 0.001). PANSS scores, insight, and depression were not significantly associated with ToM (*p* > 0.002). Duration of illness and hospitalization were unrelated to TOM tests.

**Table 3:** Bivariate analysis taking the theory of mind scale as the dependent variable among the entire sample.

	First order false belief (TOM-15)	p-value	Second order false belief (TOM-15)	p-value	Comprehension (TOM-15)	p-value
	Mean ± SD		Mean ± SD		Mean ± SD	
Participants status						
Schizophrenia patients	4.43 ± 2.11	**<0.001**	3.90 ± 1.87	**<0.001**	10.63 ± 3.52	**<0.001**
Healthy control	6.91 ± 1.56		5.46 ± 1.44		13.51 ± 1.81	

Sex						
Male	4.98 ± 2.20	0.340	4.11 ± 2.01	0.025	11.04 ± 3.91	0.020
Female	5.31 ± 2.36		4.75 ± 1.64		12.21 ± 2.27	

Education level						
Complementary	4.45 ± 2.10	0.066	3.96 ± 1.93	0.414	10.93 ± 3.28	0.577
Secondary	5.15 ± 2.26		4.39 ± 1.75		11.38 ± 3.54	
University	5.52 ± 2.29		4.36 ± 2.19		11.80 ± 3.51	

Marital status						
Single/widowed/divorced	4.94 ± 2.25	0.042	4.23 ± 1.94	0.067	11.33 ± 3.48	0.141
Married	5.82 ± 2.23		4.90 ± 1.62		12.30 ± 2.78	

Family history of psychiatric illness						
Yes	4.72 ± 2.25	0.376	3.85 ± 2.04	0.106	10.51 ± 3.95	0.084
No	5.05 ± 2.19		4.39 ± 1.81		11.59 ± 3.14	


	Correlation coefficient	p-value	Correlation coefficient	p-value	Correlation coefficient	p-value
Age	−0.124	0.116	−0.091	0.268	−0.164	0.053

Values marked in bold indicate significant ***p***-values after Bonferroni correction ***p*** < 0.002.

**Table 4:** Correlation analysis between the theory of mind and the quantitative scales among patients with schizophrenia.

	First order false belief (TOM-15)	p-value	Second order false belief (TOM-15)	p-value	Comprehension (TOM-15)	p-value
	Correlation coefficient		Correlation coefficient		Correlation coefficient	
BACS total score	0.318	**0.001**	0.254	0.014	0.331	**0.002**
Verbal memory	0.172	0.087	0.207	0.046	0.252	0.019
Working memory	0.220	0.028	0.213	0.040	0.147	0.177
Motor speed	0.331	**0.001**	0.209	0.044	0.293	0.006
Verbal fluency	0.282	0.005	0.236	0.023	0.319	0.003
Attention and speed of information processing	0.218	0.030	0.241	0.020	0.362	**0.001**
Executive function	0.149	0.138	−0.001	0.994	−0.027	0.808
PANSS total score	0.029	0.743	−0.014	0.880	0.130	0.179
Positive PANSS scale	0.047	0.593	0.089	0.339	0.112	0.250
Negative PANSS scale	0.002	0.981	−0.069	0.459	0.098	0.315
General psychopathology PANSS scale	0.022	0.807	−0.039	0.678	0.109	0.262
Insight Scale for psychosis	0.086	0.332	−0.015	0.875	−0.020	0.841
Depression scale	0.073	0.407	−0.105	0.258	0.051	0.598
Duration of illness	−0.048	0.587	0.008	0.930	−0.038	0.699
Duration of hospitalization	−0.125	0.155	−0.066	0.481	−0.173	0.073
Number of hospitalization	−0.042	0.637	−0.086	0.354	−0.262	0.006

Values marked in bold indicate significant p-values after Bonferroni correction p < 0.002.

Table 5 shows the association between TOM task performances according to Antipsychotic medication use in patients with schizophrenia. No significant differences were observed in the first- and second-order false belief subtests for all antipsychotics use (*p* > 0.05 for all). No significant associations were found with chlorpromazine equivalent dose (p > 0.05).

**Table 5:** Association between the theory of mind and the antipsychotic medication used by patients with schizophrenia.

		First-order false belief (TOM-15)	p-value	Second-order false belief (TOM-15)	p-value	Comprehension (TOM-15)	p-value
		Mean ± SD		Mean ± SD		Mean ± SD	
First generation antipsychotics							
Pimozide	Yes	5.83 ± 2.13	0.097	4.66 ± 2.42	0.311	12.00 ± 3.00	0.501
	No	4.36 ± 2.10		3.86 ± 1.85		10.60 ± 3.54	
Haloperidol	Yes	4.29 ± 2.10	0.372	3.69 ± 1.88	0.123	10.40 ± 3.48	0.411
	No	4.63 ± 2.13		4.23 ± 1.84		10.97 ± 3.61	
Zuclopenthixol	Yes	4.29 ± 1.94	0.723	4.00 ± 1.65	0.790	9.13 ± 4.26	0.025
	No	4.46 ± 2.16		3.88 ± 1.93		11.02 ± 3.23	
Chlorpromazine	Yes	4.84 ± 1.99	0.144	3.97 ± 2.17	0.801	10.65 ± 2.63	0.974
	No	4.25 ± 2.15		3.87 ± 1.74		10.63 ± 3.86	

Second generation antipsychotics							
Risperidone	Yes	4.47 ± 2.55	0.906	4.22 ± 2.40	0.473	11.33 ± 4.06	0.317
	No	4.42 ± 2.02		3.83 ± 1.74		10.47 ± 3.39	
Clozapine	Yes	4.57 ± 2.46	0.650	4.41 ± 1.83	0.136	10.08 ± 3.94	0.400
	No	4.38 ± 1.99		3.77 ± 1.87		10.78 ± 3.41	
Olanzapine	Yes	4.57 ± 1.98	0.743	4.00 ± 1.32	0.825	11.41 ± 2.73	0.328
	No	4.40 ± 2.14		3.89 ± 1.96		10.49 ± 3.65	
Quetiapine	Yes	4.50 ± 2.07	0.935	5.50 ± 1.76	0.032	11.16 ± 2.48	0.708
	No	4.42 ± 2.12		3.81 ± 1.85		10.60 ± 3.58	


	First-order false belief (TOM-15)	p-value	Second-order false belief (TOM-15)	p-value	Comprehension (TOM-15)	p-value
	Correlation coefficient		Correlation coefficient		Correlation coefficient	
Chlorpromazine equivalent dose	0.149	0.090	−0.058	0.534	0.012	0.899

Values marked in bold indicate significant ***p***-values after Bonferroni correction ***p*** < 0.002.

**Table 6:** Multivariable linear regression analysis among patients with schizophrenia.

Model 1 taking the First order false belief (TOM-15) as the dependent variable.					
	Unstandardized beta	Standardized beta	p-value	Confidence interval	
				Lower bound	Upper bound
Sex	−0.568	−0.145	0.173	−1.391	0.255
Age	−0.036	−0.138	0.185	−0.089	0.017
Family history of psychiatric illness	−0.629	−0.165	0.106	−1.394	0.136
Marital status	−1.736	−0.183	0.072	−3.633	0.161
Education level	−0.153	−0.054	0.611	−0.747	0.442
Depression scale	0.019	0.045	0.656	−0.066	0.104
Insight scale	0.035	0.039	0.719	−0.157	0.227
Positive PANSS scale	−0.003	−0.012	0.928	−0.059	0.054
Negative PANSS scale	0.005	0.021	0.840	−0.048	0.058
General psychopathology PANSS scale	−0.003	−0.028	0.835	−0.037	0.030
BACS total	0.011	0.296	0.009	0.003	0.020


Model 2 taking the Second order false belief (TOM-15) as the dependent variable.					
	Unstandardized beta	Standardized beta	p-value	Confidence interval	
				Lower bound	Upper bound
Sex	1.066	0.279	0.012	0.240	1.892
Age	0.017	0.066	0.536	−0.037	0.070
Family history of psychiatric illness	−0.248	−0.067	0.522	−1.014	0.519
Marital status	−0.103	−0.012	0.912	−1.953	1.747
Education level	−0.406	−0.148	0.195	−1.023	0.212
Depression scale	−0.098	−0.241	0.025	−0.182	−0.013
Insight scale	−0.089	−0.100	0.375	−0.287	0.109
Positive PANSS scale	0.039	0.176	0.173	−0.018	0.097
Negative PANSS scale	−0.007	−0.029	0.793	−0.064	0.049
General psychopathology PANSS scale	−0.022	−0.181	0.190	−0.055	0.011
BACS total	0.012	0.333	0.004	0.004	0.021


Model 3 taking the comprehension (TOM-15) as the dependent variable.					
	Unstandardized beta	Standardized beta	p-value	Confidence interval	
				Lower bound	Upper bound
Sex	1.317	0.190	0.089	−0.203	2.838
Age	−0.052	−0.113	0.293	−0.150	0.046
Family history of psychiatric illness	−1.094	−0.157	0.137	−2.544	0.356
Marital status	−3.071	−0.193	0.073	−6.437	0.295
Education level	−0.820	−0.160	0.164	−1.983	0.342
Depression scale	−0.047	−0.064	0.553	−0.206	0.111
Insight scale	0.080	0.048	0.681	−0.306	0.466
Positive PANSS scale	0.012	0.029	0.822	−0.095	0.119
Negative PANSS scale	0.025	0.054	0.628	−0.078	0.128
General psychopathology PANSS scale	0.020	0.087	0.525	−0.041	0.081
BACS total	**0.028**	**0.416**	**0.001**	**0.013**	**0.044**

Variables entered Age, gender, family history of psychiatric illness, marital status, education level, insight, PANSS scale and BACS scale.

Values marked in bold indicate significant p-values after Bonferroni correction p < 0.002.

### 3.5 Multivariable analysis

Three linear regression models were performed taking the TOM subtests as the dependent variables. In the first and second models, First-order false belief and Second-order false belief were used as the dependent variables, respectively. The results showed that a higher BACS total score tended to be significantly associated with better performance on these tasks (Table 6, Model 1, model 2).

The third model taking the comprehension test (TOM-15) as the dependent variable showed that a higher cognitive function (Beta=0.028) was significantly associated with higher comprehension test scores (Table 6, Model 3).

## 4 Discussion

The findings of this study align with the extensive body of research demonstrating significant impairments in TOM among individuals with schizophrenia. Chronic patients with schizophrenia exhibited notable TOM deficits compared to healthy controls. Patients have lower scores on first-order false belief tasks, on second-order false belief tasks, and on the comprehension task than the healthy control group. These differences remained statistically significant after adjusting for potential confounders. These results support Frith's (1992) conceptualization of schizophrenia as a disorder primarily characterized by impairments in the representation of mental states, where difficulties in understanding others' mental states are a core feature of the illness (Bora et al., 2008; Frith, 2014). The moderate impairments in TOM demonstrated in the current study were less pronounced compared to those observed in previous studies (Corcoran et al., 1995; Pickup and Frith, 2001). This discrepancy may be attributable to several methodological differences, including a larger sample size compared to earlier studies. However, this explanation is tentative, further research is needed to explore the potential influence of diverse sample size on the observed TOM deficits.

Additionally, the variability in TOM deficits across studies may partly be attributed to the complex and multifaceted nature of TOM, which is often operationalized differently depending on the research focus. Some studies have concentrated on the cognitive aspects of TOM (Pickup and Frith, 2001), others on its affective components (Wu et al., 2023; Shamay-Tsoory et al., 2007) and some have examined both dimensions simultaneously (Harrington et al., 2005; Brüne, 2005; Bora et al., 2008). This lack of standardization in TOM assessments, along with differences in task formats such as verbal narratives, visual images, or short films, and variations in response types like open-ended versus multiple-choice questions, may contribute to inconsistencies in the severity of TOM impairments (Harrington et al., 2005; Brüne, 2005; Sprong et al., 2007). Furthermore, the limited availability of psychometric data regarding the reliability and validity of these tasks may further complicate the interpretation of findings, thereby limiting the ability to draw definitive conclusions about TOM deficits in schizophrenia.

Another possible explanation for the relatively less pronounced TOM impairments observed in this study may involve cultural factors. Research has previously demonstrated that social cognition, can be, to a certain degree, influenced by broader sociocultural environment in which individuals develop and interact (Lillard, 1998; Heyes and Frith, 2014). In collectivist societies such as Lebanon, where social interdependence, family cohesion, and group-oriented values are strongly emphasized, individuals may engage more consistently in structured social interactions. These culturally embedded patterns of interpersonal engagement could in fact, help support the development or preservation of certain social cognitive functions, even in the presence of chronic psychiatric illness. However, this explanation remains speculative and requires further cross-cultural research to explore the extent to which sociocultural factors influence TOM performance in schizophrenia.

Moving on, the present study identified heterogeneity in TOM performance among Lebanese patients with chronic schizophrenia, particularly when comparing first-order and second-order false belief tasks. Participants demonstrated superior performance on first-order tasks relative to second-order tasks. This finding aligns with previous research, which suggests that second-order TOM tasks are inherently more complex (Harrington et al., 2005; Brüne, 2005; Sprong et al., 2007; Bora et al., 2008). These tasks not only require basic perspective-taking but also necessitate the ability to process and reconcile multiple, sometimes conflicting, viewpoints regarding others' beliefs and intentions. Such increased cognitive complexity places greater demands on higher-order cognitive functions, including working memory, attention, and executive functioning (Brüne, 2005; Corcoran et al., 1995; Ventura et al., 2013; Fanning et al., 2012). Consistent with this, our study found significant correlations between second-order TOM performance and several cognitive domains, such as verbal fluency, verbal memory, working memory, attention, and processing speed, further supporting the idea that more complex TOM tasks may be disproportionately affected by cognitive deficits in schizophrenia.

Additionally, the study observed relatively high comprehension scores, indicating that participants could understand basic social scenarios. This finding is consistent with previous research by Champagne-Lavau and Charest (2015) and Green et al. (2008), which showed that individuals with schizophrenia often possess a reasonable ability to interpret simple contextual cues. However, despite this, they struggle with understanding the mental states and intentions of other. These results suggest that while individuals with schizophrenia may comprehend basic social information, they face significant difficulties with more advanced aspects of TOM, such as interpreting others' beliefs and intentions.

Frith (1992) proposed a symptom-specific hypothesis, suggesting that deficits in TOM in schizophrenia are directly associated with specific symptom dimensions (Frith, 2014). According to this hypothesis, negative symptoms, such as apathy, hinder the understanding of both self and others' mental states, while positive symptoms, like delusions, contribute to misinterpretations of social interactions (Frith and Frith, 2003; Frith, 2014). However, the present study did not find significant correlations between either positive or negative symptoms and TOM performance, thereby challenging Frith's theoretical framework. In fact, support for Frith's model has been inconsistent in the literature, with some studies identifying associations between symptom dimensions and TOM impairments (Corcoran et al., 1995; Pickup and Frith, 2001; Wu et al., 2023; Abdel-Hamid et al., 2009; Mazza et al., 2012), while others report no such associations. This variability may be attributed to differences in the methodological approaches used to classify and measure clinical symptoms (Fernandez-Gonzalo et al., 2013; Wang et al., 2013; Li et al., 2017). Studies often employ varying symptom categorization models, such as Frith's hierarchical neuropsychological model (Frith, 1996), Liddle's Three-Factor Division Model (Bassett et al., 1994), or the PANSS (Harrington et al., 2005). Furthermore, the heterogeneity of symptom expression in schizophrenia, with patients displaying varying combinations of positive and negative symptoms, may contribute to the observed inconsistency in findings. This overlap complicates the establishment of clear symptom-specific relationships with TOM deficits, making it difficult to apply Frith's model uniformly across different subgroups of patients (Harrington et al., 2005; Sprong et al., 2007; Bora et al., 2008).

Moving on to neurocognition and TOM performance. This study found weak to moderate correlations between neurocognitive functioning, as assessed by the BACS total score, and TOM performance, suggesting that neurocognitive factors may influence social cognitive processes in schizophrenia. Specifically, significant positive correlations were found between the BACS total score and TOM tasks: first-order false belief, second-order false belief, and comprehension. These results indicate that individuals with higher neurocognitive abilities tend to perform better on TOM tasks. The findings are consistent with previous research, including Fanning et al. (2012) and Ventura et al. (2013), which also highlighted the relationship between neurocognition and TOM in schizophrenia. While the current results support the idea that TOM deficits may reflect broader neurocognitive impairments, the exact nature of the relationship between these domains remains unclear. Further research is necessary to elucidate the hierarchical interplay between neurocognitive functions and social cognition in schizophrenia (Thibaudeau et al., 2020).

Interestingly, the present study revealed a gender-related difference in cognitive TOM, with female participants performing better than males on both second-order TOM and comprehension tasks. This finding is consistent with previous research indicating that females tend to outperform males on social cognitive measures (Abu-Akel and Bo, 2013), a pattern observed in both clinical and non-clinical populations. Such differences have been attributed to greater female capacity for emotional understanding and perspective-taking. Additionally, gender-related advantages may reflect better premorbid functioning among women with schizophrenia, including stronger social relationships and higher levels of attention, education, and occupational attainment (Canuso and Pandina, 2007). Additionally, the higher comprehension scores observed among females in the current study may also relate to broader cognitive strengths in domains such as language, memory, and executive function, which may facilitate more effective TOM performance (Han et al., 2012).

Lastly, the present study did not found a correlation between depression scores and TOM performance. This finding implies that within this sample depressed symptoms did not seem to affect the capacity to comprehend the mental states of others. Our results suggest that TOM deficits in schizophrenia may be largely independent of depressive symptoms, despite some prior studies reporting associations between affective symptoms and social cognition impairments. This suggests that rather than comorbid affective disturbances, TOM deficits may be more strongly associated with the core characteristics of schizophrenia. Additionally, depressive symptoms like emotional blunting, apathy, and social withdrawal can overlap with negative symptoms of schizophrenia, further hindering the ability to engage in social cognitive tasks.

### 4.1 Limitations of the study

This study had several methodological limitations that need to be acknowledged. Firstly, it is important to point out the lack of validation of the Arabic translation of the TOM 15 false belief task. This introduces some uncertainty regarding the reliability and accuracy of the measurement tool in our specific population. Secondly, the study recruited multiple examiners, all with psychology or psychiatry training and credentials to conduct the interviews during data collection. The involvement of multiple examiners may have introduced ‘inter-rater biases’, where different examiners might influence the responses of participants differently. This potential risk might have introduced inconsistencies with the administration or interpretation of the scales. Inter-rater reliability was not formally assessed in this study. Lastly, this study was conducted using a cross-sectional research design, which comes with inherent limitations. Specifically, this design restricts the ability to establish causal relationships, because it captures data and a specific moment of time.

### 4.2 Future recommendations

In regard to the measurement tools for TOM, we note a pressing need for studies evaluating the psychometric and cultural validity of the ToM-15 scale in the Lebanese general population as well as in patients with Schizophrenia. Additionally, since this study employed a false belief task, which primarily assesses the cognitive aspect of TOM, it would be interesting to assess the affective component of TOM in the Lebanese schizophrenia population. This could provide valuable insights into whether there are differences in the severity of TOM impairments between the cognitive and affective components. Finally, we suggest that intervention-based studies using various psychotherapies or pharmacotherapies for depression and neurocognitive impairment in Schizophrenic patients could be highly interesting, with a pre and post-intervention assessment of the TOM abilities.

## 5 Conclusion

In conclusion, this study provided valuable insights into TOM impairments in a sample of chronic Lebanese in-patients diagnosed with schizophrenia. The results revealed significant TOM impairments in patients with schizophrenia as compared to healthy controls, with greater difficulties observed in second-order false belief tasks. No significant correlations were found between TOM performance and clinical symptom severity, possibly due to the disorder's heterogeneity. Neurocognitive performance showed weak to moderate associations with TOM abilities, suggesting a partial influence. More longitudinal studies are needed to further understand the association between impaired TOM and other factors such as depression, neurocognition, and schizophrenia symptom severity.

The following is the supplementary data related to this article.

Table S1 Comparison of cognitive performance on BACS domains between schizophrenia patients and healthy controls Table S1

## Abbreviations

TOM: Theory of Mind
HPC: Psychiatric Hospital of the Cross
TOM-15: Theory of Mind-15
BACS: Brief Assessment of Cognition in Schizophrenia
PANSS: Positive and Negative Syndrome Scale
CDSS: Calgary Depression Scale for Schizophrenia
IS: Insight Scale for Psychosis
SPSS: Statistical Package for the Social Sciences
ANOVA: Analysis of variance
SD: standard deviation

## CRediT authorship contribution statement

**Marie Ghosn:** Writing – original draft, Project administration. **Chadia Haddad:** Writing – review & editing, Validation, Formal analysis, Conceptualization. **Jean-Marc Rabil:** Writing – review & editing, Project administration. **Georges Haddad:** Writing – review & editing, Validation, Supervision.

## Consent for publication

Not applicable.

## Funding

No funding was received for conducting this study.

## Declaration of competing interest

The authors have no competing interests to declare that are relevant to the content of this article.

## Data Availability

The datasets generated during and/or analyzed during the current study are available from the corresponding author on reasonable request.
