# Supplementary material for: Exploring theory of mind abilities in Lebanese chronic patients with schizophrenia: A cross-sectional study
Source: Schizophr Res Cogn. 2025 Aug 6;42:100385. doi: 10.1016/j.scog.2025.100385 (PMC12346024; doi:10.1016/j.scog.2025.100385)
Supplement: Table S1 — Comparison of cognitive performance on BACS domains between schizophrenia patients and healthy controls [file mmc1.docx]

**Supplementary material**

| **Table S1: Comparison of Cognitive Performance on BACS Domains Between Schizophrenia Patients and Healthy Controls** | | | |
| --- | --- | --- | --- |
|  | **Schizophrenia patients (N=146)** | **Healthy control**  **(N=50)** | **p-value** |
|  | **Mean ± SD** | **Mean ± SD** |  |
| **BACS total score** | 116.90 ± 48.62 | 221.34 ± 42.74 | <0.001 |
| Verbal memory | 23.35 ± 11.30 | 41.09 ± 9.66 | <0.001 |
| Working memory | 11.05 ± 5.30 | 20.15 ± 4.96 | <0.001 |
| Motor speed | 39.55 ± 18.47 | 68.50 ± 15.64 | <0.001 |
| Verbal fluency | 21.69 ± 8.83 | 34.59 ± 10.42 | <0.001 |
| Attention and speed of information processing | 12.69 ± 11.54 | 39.00 ± 10.54 | <0.001 |
| Executive function | 8.55 ± 7.24 | 18.00 ± 4.26 | <0.001 |
